# Perceptions of family, community and religious leaders and acceptability for minimal invasive tissue sampling to identify the cause of death in under-five deaths and stillbirths in North India: a qualitative study

**DOI:** 10.1186/s12978-021-01218-4

**Published:** 2021-08-04

**Authors:** Manoja Kumar Das, Narendra Kumar Arora, Gurkirat Kaur, Prikanksha Malik, Mahisha Kumari, Shipra Joshi, Reeta Rasaily, Harish Chellani, Harsha Gaikwad, Pradeep Debata, K. R. Meena

**Affiliations:** 1 The INCLEN Trust International, New Delhi, 110020 India; 2 Division of Reproductive Biology Maternal and Child Health, Indian Council of Medical Research, New Delhi, 110029 India; 3 Department of Pediatrics, Safdarjung Hospital and Vardhman Mahavir Medical College, New Delhi, 110029 India; 4 Department of Obstetrics and Gynaecology, Safdarjung Hospital and Vardhman Mahavir Medical College, New Delhi, 110029 India

**Keywords:** Minimal invasive tissue sampling, Cause of death, Neonates, Child, Stillbirth, Formative research, Parents, Community, Religious leaders, India

## Abstract

**Background:**

Minimal invasive tissue sampling (MITS) has emerged as a suitable alternative to complete diagnostic autopsy (CDA) for determination of the cause of death (CoD), due to feasibility and acceptability issues. A formative research was conducted to document the perceptions of parents, community and religious leaders on acceptability of MITS.

**Methods:**

This qualitative study was conducted at and around the Safdarjung Hospital, Delhi, India. Participants for in-depth interview included the parents who had either child or neonatal death or stillbirth and the key community and religious representatives. The focus group discussions (FGDs) involved community members. Process of obtaining consent for MITS was observed. Data were analyzed inductively manually for emerging themes and codes.

**Results:**

A total of 104 interviews (parents of deceased children, neonates or stillbirths, n = 93; community members, n = 8 and religious leaders, n = 7), 8 FGDs (n = 72) were conducted and process of obtaining consent for MITS (n = 27) was observed. The participants were positive and expressed willingness to accept MITS. The key determinants for acceptance of MITS were: (1) understanding and willingness to know the cause of death or stillbirth, (2) experience of the healthcare received and trust, (3) the religious and sociocultural norms. Parents and community favored for MITS over CDA when needed, especially where in cases with past stillbirths and child deaths. The experience of treatment, attitude and communication from healthcare providers emerged as important for consent. The decision making process was collective involving the elders and family. No religious leader was against the procedure, as both, the respect for the deceased and need for medical care were satisfied.

**Conclusions:**

Largely, MITS appeared to be acceptable for identifying the causes of child deaths and stillbirths, if the parents and family are counseled appropriately considering the sociocultural and religious aspects. They perceived the quality of care, attitude and communication by the healthcare providers as critical factors for acceptance of MITS.

**Supplementary Information:**

The online version contains supplementary material available at 10.1186/s12978-021-01218-4.

## Plain language summary

India tops the chart of childhood deaths and stillbirths globally. The causes of many childhood deaths and stillbirths remain unknown or ill-defined in India. For planning the health policies, program and clinical practices, the cause of death (CoD) and stillbirth are primarily determined by verbal autopsy (VA) method, which has several inherent limitations. The acceptance and implementation of complete diagnostic autopsy (CDA) or full autopsy for CoD determination is limited. Minimally invasive tissue sampling (MITS) is a suitable alternative to full autopsy for CoD determination. MITS has been found to be acceptable and being implemented in some low and middle income countries. Considering the multi-religion and sociocultural contexts of India, an understanding about the perceptions, practices, acceptance and barriers among the parents, community members and religious leaders regarding MITS was necessary for designing appropriate communication strategies and materials for consent. A qualitative study was undertaken in New Delhi, India using in-depth interviews with the parents of deceased children and stillbirths, community leaders, community health workers and religious leaders and focus group discussions with the community members and observation of obtaining consent for MITS. The parents and community members were positive about MITS and expressed willingness to accept it over full autopsy to know the CoD and stillbirths. The factors like past stillbirths and child deaths, experience of the healthcare received and trust, the religious and sociocultural norms emerged as important determinants for acceptance of MITS. The religious leaders were also positive about MITS if needed for treatment and benefit of the family.

**Supplementary Information:**

The online version contains supplementary material available at 10.1186/s12978-021-01218-4.

## Background

Approximately 5.3 million under-five children died globally in 2018, about 15,000 deaths daily [1]. Infant (74%) and neonatal deaths (46%) constituted the major share of the under-five deaths [1]. An equal number of stillbirths also occur annually [2, 3]. India with 882,000 under-five deaths in 2018 was the largest contributor globally [1]. During 2015, about 0.59 million stillbirths occurred in India [4]. Although there has been tremendous progress, the decline in neonatal deaths and stillbirths have been slower. Most of these infant and early childhood deaths are preventable through cost-effective and basic quality-delivered interventions [5]. The National Health Policy, India (2017) aims to reduce the under-five mortality from 43 to 23 and neonatal mortality from 24 to < 10 per 1000 livebirths by 2025 [6]. To accelerate the under-five mortality reduction, better characterization of the cause of death (CoD) in children is critical. The CoD labelling is a challenge in India, as many children die outside hospitals and a sizable proportion of those who reach a healthcare provider, do not have adequate documentation of CoD [7, 8]. Even among those who die at health facilities, the CoD in death certificates is often incomplete and poorly documented, when coexisting illnesses are there, which make usage for appropriate intervention challenging [9–11]. The practice of complete diagnostic autopsy (CDA) to identify the CoD is dismal due to sociocultural and religious beliefs, technical, financial and infrastructure limitations [12–16]. The CoDs available and used in India and many developing countries are based on verbal autopsy (VA), which have several limitations [17–20]. The non-specific clinical features in newborns and infants make the identification of the exact CoD difficult with the VA method. The VA is not useful for stillbirths.

Absence of the true CoDs compromises effective policy planning, program implementation and thereby the effectiveness. With the impracticality of the CDA and limitations of VA, alternate procedures for CoD identification are needed. The post-mortem minimally invasive tissue sampling (MITS) has emerged as a suitable alternative, which involves detailed examination, imaging and collects tissue samples from targeted organs for histopathologic, microbiological and other appropriate investigations [21–23]. MITS is less invasive, non-disfiguring and time and cost efficient than CDA, but has comparable outcome [24–26]. The CDA is rarely performed in India for CoD identification, unless legally mandated and poorly accepted by the public [27, 28]. The contextual factors and challenges that influence consent and conduct of MITS have been reported from other countries [29–31], their relevance may not be generalizable in view of sociocultural, and religious contexts.

As part of the Child Health and Mortality Prevention Surveillance Network project, a pilot project was initiated in India to document the feasibility, acceptability, capacity building structures and standardization of the sample collection and processing to determine the CoD. This project targeted conducting MITS for under-five deaths (including newborns) and stillbirths. As MITS is relatively new for Indian context, to understand the perceptions, practices, contextual factors and barriers acceptability and obtaining consent for MITS, this formative research was conducted.

## Methods

This study was conducted in two phases during September 2018 to April 2019. The phase 1 (September 2018–February 2019) documented the perception, sociocultural and religious norms, and acceptance for MITS. The MITS was initiated since March 2019. The Phase 2 (March–April 2019) documented the process and initial experience of obtaining consent for MITS. The detailed protocol (Phase 1 and 2) has been published earlier [32].

### Study design and setting

This formative research used exploratory qualitative research design and was conducted in New Delhi, India around Safdarjung Hospital (SJH), where the MITS pilot study is being undertaken. The in-depth interviews (IDIs) and focus group discussions (FGDs) were conducted at the respective locations of stakeholders.

### Study participants

For the phase I, three categories of participants were included: (i) parents and family members of under-five children, neonates and stillbirths who died in SJH in last 6–8 weeks; (ii) community members from the SJH catchment area; and (iii) religious leaders and burial site representatives. The Table 1 shows the participants in the study. Parents of the children who died and stillbirths that occurred at SJH were contacted over phone and/or by home visit after 6–8 weeks of the unfortunate event. Families from different religions and geographies of Delhi with both parents available were purposively identified and approached for consent. The parents from outside Delhi were excluded. The community members for IDIs and for FGDs were purposively identified from different areas. For the phase II, the participants included parents and family members of deceased children and stillbirths at SJH, who were approached for consent to conduct MITS.

**Table 1:** The stakeholders participated in the in-depth interviews, focus group discussions and observations

Sl	Stakeholder category	Number
*1*	*In-depth interviews (IDIs)*	
1.1	Parents and family members (of deaths/stillbirths occurred 6–8 weeks ago)	93
	Deceased children (> 1 month–5 years) (25 parents of 13 children)	25
	Deceased neonates (< 1 month) (24 parents of 12 neonates)	24
	Stillbirths (44 participants of 22 stillbirths)	44
1.2	Community members	8
	Influential community leaders	4
	Anganwadi workers, community maternal and child nutrition workers	2
	ASHAs, community maternal and child health workers	2
1.3	Religious leaders (Hindu, Muslim, Christian, Sikh)	4
1.4	Burial site representatives (Hindu/Sikh, Muslim, Christian)	3
*2*	*Focus Group Discussions (FGDs)*	
2.1	Community participants	72
	Fathers (of children aged < 5 years) (2 FGDs)	18
	Mothers (of children aged < 5 years) (2 FGDs)	19
	Grand-fathers (2 FGDs)	17
	Grand-mothers (2 FGDs)	18
*3*	*Observation of obtaining consent for MITS*	
3.1	Parents approached for consent	27
	Deceased child (> 1 month–5 years)	5
	Deceased neonate (< 1 month)	16
	Stillbirths	6

### Data collection

Different semi-structured guides for IDIs and FGDs were developed according to the types of participants (Additional file 1). The IDI and FGD guides with open-ended questions explored participant’s understanding about the cause(s) of death in children or stillbirth followed by perceived benefit, concerns, challenges and acceptance of MITS from their sociocultural and religious perspectives. The IDI and FGDs guides were pilot tested and refined based on the findings. Two pairs of female researchers trained in qualitative research and interviewing conducted the interviews at homes or places convenient for participants. The interviews with mother and father of the child/stillborn were conducted separately. The FGDs had 8–11 participants from similar category and were conducted at convenient and neutral venues in the community. These were facilitated by the lead investigator (MKD, male) experienced in conducting IDI, FGD and qualitative research supported by the four female researchers. During the IDIs and FGDs, presence of other person was avoided as far as possible. For few IDIs, presence of other family member was experienced, but the IDI was interrupted briefly and was resumed when the other person left the IDI venue. The IDIs and FGDs were conducted in local language (Hindi) and were audio-recorded. Detailed field notes were taken capturing the verbal and non-verbal expressions. The median time taken for the IDI and FGDs was about 60 (45–90) min. One researcher observed the counseling for MITS after death/stillbirth in the ward or outside the ward. The researcher didn’t participate in the counseling or consenting process and no interview with parents/family or audio-recording was done, considering the sensitivity of the situation and process. The field notes of the conversations were captured.

### Data handling and analysis

The audio recordings and field notes were transcribed verbatim in local language by two researchers followed by translation into English. Two other team members checked the quality of the transcriptions with the audio-records for completeness and correctness. The observation field notes were transcribed and translated. The data was entered using INCLEN Qualitative Data Analysis Software (IQDAS), which allows data entry, organization and retrieval for analysis in Indian languages and English. The data entered were checked for correctness and completeness by another team member. The data entered were saved into the server and backed up on daily basis. The transcripts were read by two researchers independently several times using the IQDAS. The data was analyzed inductively following the steps: free listing, domain identification, coding, and cross tabulation. The emerged codes and themes were discussed by the team and the discrepancies were resolved periodically. The IDIs, FGDs and observations were coded separately. These codes were reviewed to identify the linkages (axial coding) and group into fewer categories (selective coding) and assemble under key themes. The transcript reading, coding and thematic summarization was a reiterative process till the investigators agreed on the final framework. The themes across the participant categories and methods were triangulated to check consistencies and differing perspectives. The findings were expressed in semi-quantitative format using qualifiers: very few (< 10%), some (10–24%), about half (25–49%), majority (50–75%), most (76–89%) and almost all (> 90%).

### Ethical considerations

The participants were recruited after obtaining written informed consent including permission for audio recording and use of anonymized quotes. Confidentiality and anonymity of participants were assured from the research team. For the FGDs, the participants were requested not to reveal the information shared by any of the participant during the discussion to any other person. For the observations, verbal consent was obtained. Ethical approval for the study was obtained from all the participating institute ethics committees.

## Results

This study included 93 parents of 25 deceased children (neonates, n = 12 and > 1 month, n = 13, participants, n = 49) and 22 stillbirths (n = 44) from different religions (Hindus, n = 26; Muslims, n = 19 and Christians, n = 2). The other participants included community members (n = 4), religious and burial functionaries (n = 7) and community health functionaries (n = 4). Eight FGDs were conducted with 72 participants (8–10 per FGD). The demographic information about the IDI and FGD participants are given in Table 2.

**Table 2:** Sociodemographic parameters of the participants

Sl no.	Parameters	Results
*1*	*In-depth interviews (IDIs)*	
1.1	Age in years, median (IQR)	
	Mother (n = 47)	25 (22–30)
	Father (n = 46)	29.5 (27–34)
1.2	Religion (n = 47)	
	Hindu	27 (57.4)
	Muslim	18 (38.3)
	Christian	2 (4.2)
1.3	Mother’s literacy (n = 47)	
	< 5th standard	11 (23.4)
	6th–10th standard	18 (38.3)
	> 10th standard	18 (38.3)
1.4	Father’s literacy (n = 46)	
	< 5th standard	7 (15)
	6th–10th standard	20 (42.5)
	> 10th standard	20 (42.5)
1.5	Mother’s occupation (n = 47)	
	Housewife	46 (98)
	Working (skilled worker)	1 (2)
1.6	Father’s Occupation (n = 46)	
	Skilled worker	21 (45.6)
	Self-employed or business	21 (45.6)
	Daily wage laborer	4 (8.7)
*2*	*Focus group discussions (FGDs)*	
2.1	Age in years, median (IQR) (n = 72)	
	Fathers (of children aged < 5 years) (2 FGDs)	28 (25–30)
	Mothers (of children aged < 5 years) (2 FGDs)	26 (25–30)
	Grand-fathers (2 FGDs)	58 (53–62)
	Grand-mothers (2 FGDs)	58 (52.5–61)

### Perceived acceptance and consent for MITS

*Acceptance and consent for MITS:* Most of the parents of deceased children had positive opinion about MITS and the purpose, which can assist to know the CoD. Similarly, most of the couples with stillbirth also expressed willingness for MITS to know the cause. Very few of the parents expressed their descent for potential MITS or were indecisive and remained silent. While few parents those who were not willing for MITS mentioned reasons including no-benefit as it may not return the baby back to life, religious norms and few didn’t mention any reason.

“This technique is right at least it would prevent cutting and disfigurement of the body.” (Mother of a deceased child)

“We would have agreed for this (MITS) test, by this at least we would have known what went wrong, what negligence happened. Also, we would know what doctors could not control and death happened.” (Father of a deceased neonate).

“We would not do it. How old was our baby? Anyway, the baby was no more. After that if such things were done, it would have been a torture for the baby. We would have felt bad” (Husband of pregnant women who had stillbirth).

On analysis, we identified four overarching themes comprising of factors that potentially influence the acceptance of MITS by parents: (1) knowledge and desire to know the causes of death or stillbirth; (2) knowledge about methods for identifying the causes of death and stillbirth; (3) sociocultural factors and decision making dynamics; and (4) healthcare provider and facility level factors. The findings along with the illustrative statements from participants are presented below.

### Knowledge and desire to know cause of death and stillbirth

*Knowledge about the cause of death or stillbirth:* Majority of the parents of children who died reported that the doctors conveyed the causes of death. While some of the parents couldn’t recall the cause, some expressed that no cause was conveyed to them. Some parents expressed that they couldn’t understand the cause mentioned and few didn’t agree with the causes conveyed. Few of the parents felt that the death declaration was quiet sudden or unanticipated without any prior clear information about the status of the child and worsening in the condition. Some of the parents recalled that some cause was explained to them at the time of death declaration, but they cannot recall, as the mental status was different at that time. Majority of the parents with stillbirths couldn’t either recall the definite cause or understand the cause told to them.

“No one told us the about the cause of death at that time. They only told that the blood vessel in child’s brain has burst. There was bleeding; it first started from mouth and then from the nose. We should know the reason. When our child was normal, how did that happen?” (Mother of the deceased child).

*Willingness to know the cause of death or stillbirth:* Most parents expressed their keenness to know the cause of death or stillbirth and perceived that some preventive measures could be taken for future pregnancy and/or other alive children. Most of the parents were unclear about the cause of stillbirth even after they followed regular pregnancy care and investigations suggested by the doctors and the pregnancy and labour progressed uneventfully. The parents who experienced multiple pregnancy losses or stillbirth or child death, strongly favoured MITS. The parents of the children who were not clear about the definite cause of the illness and death or who did not agree with the cause(s) mentioned by the doctors voiced for MITS. More mothers of the deceased children and stillborn were willing to know the CoD than the fathers. The parents of few deceased children and stillbirths were not willing to know the CoD and considered it as act of god and useless, as the baby would not return back to life.

“This is important for future planning and to know whether there was anything wrong in the treatment” (Mother of the deceased child)

“There is a great desire to know. I feel that if the cause is known, we can avoid in future. I am very scared for next pregnancy” (Mother who had stillbirth)

### Knowledge about methods for identifying the cause of death and stillbirth

*Knowledge and perceptions about methods for CoD:* Almost half of the respondents were aware about the CDA for knowing the CoD in cases of unnatural deaths and accidents. Some perceived that the organs were removed for transplantation. Few respondents expressed their unpleasant experience with CDA in their family or neighbourhood including the disfigurement, handover of poorly stitched bodies and time delay. Almost all were vocal against the CDA and especially for the children and stillbirths, citing the emotional and religious reasons (especially Muslim parents). The perceptions were similar across the respondents from different socioeconomic and professional strata.

“Post-mortem of child cannot take place. It is not allowed in our religion also. The person who has an accident has a post mortem in the hospital itself. If his heart is alive, so doctors take out all organs by doing post mortem.” (Mother of the deceased child).

“It (post-mortem) is forbidden in our religion.” (Mother of the deceased child)

*Perceived benefits of MITS and reasoning:* Most of the parents of deceased neonates, infants and stillbirths, who favoured MITS perceived it to be beneficial for their next pregnancy. The parents of older children considered potential benefits for their living children and the society. Most of the participants perceived the MITS to be more acceptable than autopsy due to avoidance of cutting, disfigurement and organ removal.

### Sociocultural factors and decision making dynamics

*Approaching and counseling for MITS:* Most of the parents suggested to approach one or two suitable male family members of the deceased child after the death declaration, when they appear to have accepted the death and approachable. Most of them told that immediately after death declaration, the parents would be in immense grief and different emotional state, which might not be suitable for any decision making.

“This technique should be explained after giving some sympathy to family, taking to side and talking and explaining it, if suddenly you will explain after death nobody will understand” (Father of deceased child).

“For this our family members like my brother could have been explained about it and how to do. Then they could have told us. We could have discussed and decided, if it was ok” (Husband of pregnant women who had stillbirth).

*Social dynamics and decision making:* According to most of the parents, the decision for MITS would be taken in consultation with the elders and close family members. About half of the parents opined that the fathers would decide in consultation with the family. The other half mentioned that the grandparents would take the decision.

“That time (during death of child) parents are not in such condition. Doctor should explain to the father or some relative then they can explain us. In hospital, if the head doctor tells us about this (MITS) it is ok” (Mother of deceased child).

“Doctor should approach the father, because ladies are weak and cry in that (after death) situation, for them it is difficult to take decision. Any senior doctor who is available at that time or the concerned person regarding this technique should approach. This should be explained verbally as at that time nobody will see any picture or video regarding this (MITS)” (Father of the deceased child).

*Community opinion:* Most of the community members favored MITS over CDA, due to avoidance of disfigurement, organ non-removal, non-violation of religious norms deviance and social acceptance. Most of them opined that parents would accept MITS for the CoD identification, when they perceive the value for future pregnancy or other children. Few mentioned the trust on the doctor, the hospital and mode of explanation to be important factors. Few were doubtful about acceptance for MITS by parents. Several respondents suggested increasing public awareness and seeking assistance from local community leaders for acceptance of MITS.

“They may agree for this (MITS), if there is no disfigurement. But for them to agree, they should be made aware through either campaign” (Community leader)

“Elders say that if postmortem is done and organ is removed, the person shall not have the organ in the next birth. But medical science does not agree with this” (Father of a child, FGD participant).

*Religious permissibility:* The opinions from the different religious leaders (Hindu, Sikh, Muslim, and Christian) had no specific objection for MITS from religious aspect, if needed to know the CoD or legal procedures. According to all the religious leaders, the human body demands respect, whether child or adult. The postmortem CDA procedure involves cutting, disfigurement and removal of organs, and thus considered disrespectful to the human body and also causes more emotional stress for the family. Regarding the MITS, opinions from all religious leaders were consistent and not against the procedure. According to them, the immediate family members may be approached for giving consent with appropriate explanation. Some suggested for campaign and messaging to increase awareness among the general public to improve the acceptance for MITS and avoid the wrong beliefs about postmortem. According to the burial site caretakers from Hindu, Sikh and Christian religions, the burial procedure would be similar irrespective of any such procedure. The Muslim burial site representative didn’t clearly opine for MITS, but was against postmortem citing the religious norms.

“Our body (mitti) is damaged when we do postmortem. If the body is cut and organs from inside are removed, it is damaged. It is not allowed” (Mother who had stillbirth)

### Healthcare provider and facility level factors

*Experience of the healthcare received:* Some parents recalled the attitude and communication by the hospital staff to be sympathetic and appropriate. Majority of the parents perceived the communication, language and body expression during the hospital stay to be inappropriate, insensitive and negative. Several respondents indicated that the healthcare providers blamed parents for the disease and the delay in care.

“No, now we don’t want to do any test. I was upset for so long for my baby, when you were supposed to do these tests then you did not do anything. Now what can you do, there is no use as my baby is no more”. (Father of deceased newborn).

“Wish, what wish? I knew it. When they started pressing hard my abdomen and putting finger inside (during delivery) and pulled the baby out. I knew all that my baby will not survive” (Mother of the deceased neonate).

*Death declaration and counselling:* Some of the parents recalled the death declaration process to be very brief and unsympathetic. They also narrated that the doctors soon left the place after death declaration. Most of the parents didn’t recall any emotional support, but few were consoled by the nurses and other staffs.

*Trust and confidence:* Most parents perceived the senior doctors to be more sympathetic and used appropriate language compared to others. Some of the parents were satisfied with the kind of treatment provided to their children. Most parents had confidence on the senior doctors and the clinical team and were willing to return back to the hospital in future. Few of the parents expressed no trust and not willing to return to the hospital again.

“Sometimes I feel that doctor has given some wrong medicines. But again I think, if they give wrong medicine, will they for only one or my child”. (Mother of deceased child)

### Observation of obtaining consent for MITS

Consent process was observed in 27 cases (stillbirths-5, neonates-16 and children-6) and consent was obtained for 12 cases (stillbirths-4, neonates-6 and children-2). Fathers (uncle in one case) consented for the MITS after consultation with elders and family, either present physically or over phone. The reasons for MITS acceptance were perceived value to know CoD and possible assistance for next pregnancy. Potential disfigurement, necessity of MITS and time needed for the procedure were the key concerns raised. Some parents or family members asked, “If post-mortem will be done?”; “Will the body be cut?”; “How much time will it take?”; “Will it identify the cause of death?” While counseling for consent, the MITS team stressed on the possible benefits and no disfigurement or cutting and use of needle for sampling. After conduct of MITS, one father verified for any disfigurement of the body. The reasons for consent refusal were dissatisfaction with the care received, low confidence on the care providers, place of residence (challenge to return during the day) and perceived no benefit.

“Will all diseases be known, will everything be known? There should not be any cutting of the baby, all be taken through injection only. (Father of stillborn, Consented)

“We already know the reason of death. We don’t want to go for sampling. I am telling you everything, none of the doctor came to check the child throughout the night. My big brother is asking me not to give any consent for any test as there is no use of it.” (Father of deceased child, Not consented).

## Discussion

This study is the first to our knowledge exploring the willingness to know the CoD and acceptability for MITS on deceased children and stillbirth in India. The CDA is not accepted by most of the families, unless for medicolegal reasons. The primarily reasons for denial are disfigurement, emotional, religious and some rumors regarding the organ retention, as reported from India and other countries [12–16, 28]. This study identified acceptability for MITS and potential influences among parents and community members in Delhi. The study observed that most of the participants were willing for MITS, if offered to know the CoD in children and stillbirths. The study also documented the determinants for acceptance, which were complex and multidimensional from a multicultural and multireligion context. The decision making for MITS were influenced by the personal and family dynamics, sociocultural and religious contexts and perceived quality of care received at the hospital.

This study found a positive attitude among parents and community members towards MITS across the religious groups was consistent with the other observations from Asian (Pakistan), African (South Africa, Gabon, Kenya, Mali, Mozambique) and European (United Kingdom, Belgium) countries [31, 33–36]. The willingness to accept MITS was due to the willingness to know the CoD and perceived benefit in future pregnancy or alive children, similar to the Pakistan study [33]. The views of parents with multiple stillbirth or child death were stronger for MITS, which was similar to the findings from Pakistan and United Kingdom [33, 35]. Adequate counseling and periodic status update during hospitalization emerged as critical to build trust and acceptance of MITS, as previous studies [29, 33, 37]. The attitude, communication and emotional support by the treating team also emerged as important factors. The decision making for MITS was dependent on the family dynamics and influence of elders and relatives. According to most of the participants, either father or father- or mother-in-law would take decision in consultation with other family members, as observed in other studies [31, 33]. Mothers were more willing to know the CoD, but decision making was limited, as observed in Pakistan, Bangladesh and South Africa [33, 38, 39]. The death declaration process, parent’s state of mind and persons present potentially influence the decision making [33, 35]. The parents and community opinions were triangulated with the observations during consent obtaining process.

Religion and cultural perspectives were considered important for MITS acceptance. A high degree of consistency across the religious groups favoring MITS was observed, but when it was perceived to be essential to know the cause of death or stillbirth that could help the family for other children or future pregnancy. The key factors like no mutilation, disfigurement, organ retrieval, and disrespect to the body were considered positive for MITS compared to the CDA. These observations were similar to the reports from Muslim and Christian dominated communities [29, 31, 33, 34, 38]. Timing of death appeared to influence MITS acceptance as the burial should be completed before end of the day [33, 37]. This is the first observation from Hindu and Sikh communities. The investigators and healthcare providers must be aware of the influencing factors for MITS acceptance, assess the situation and account them appropriately while approaching for consent. The community members suggested appropriate communication for demand generation to know the CoD to improve MITS acceptance.

The multi-religion and multi-stakeholder composition of the participants and triangulation of the perceptions during observations for obtaining consent were the strengths. The exploration of MITS acceptance hypothetically from parents who had stillbirth or child death at one hospital were the possible limitations.

## Conclusion

This study documented that MITS is likely to have higher acceptability compared to CDA for child deaths and stillbirths as an option for CoD identification. The parents would accept MITS if they receive appropriate care, communication and respectful counseling about the benefit with assurance about no disfigurement. Early engagement with the family and transparency in information sharing appeared to be key for the acceptance. Additionally, the health system and professionals should be prepared for MITS guaranteeing the necessary sensibility and human rapport. In view of promising role of the MITS in high mortality burden settings, this study contributes to the scarce body of knowledge about barriers and facilitators for implementing MITS in India.

## Supplementary Information

**Additional file 1:** The in-depth interview and focus group discussion guides used for data collection.

## Data Availability

The datasets used and analyzed during the current study are available from the corresponding author on reasonable request.

## Abbreviations

CDA: Complete diagnostics autopsy
CoD: Cause of death
FGD: Focus group discussion
IDI: In-depth interview
IQDAS: INCLEN Qualitative Data Analysis Software
MITS: Minimally invasive tissue sampling
SJH: Safdarjung Hospital
VA: Verbal autopsy
