# Supplementary material for: Perceptions of family, community and religious leaders and acceptability for minimal invasive tissue sampling to identify the cause of death in under-five deaths and stillbirths in North India: a qualitative study
Source: Reprod Health. 2021 Aug 4;18:168. doi: 10.1186/s12978-021-01218-4 (PMC8336381; doi:10.1186/s12978-021-01218-4)
Supplement: Supplementary file 1 — Additional file 1: The in-depth interview and focus group discussion guides used for data collection. [file 12978_2021_1218_MOESM1_ESM.docx]

**Key Informant In-depth interview guide**

**Parents of Deceased Newborn and Child**

1. **Basic demography**
   1. Demographic information
      1. Age
      2. Education
      3. Type of residence
      4. Religion
      5. Origin residence
   2. Family structure
      1. Composition- members
      2. Number of children
   3. Occupation
      1. Mother
      2. Father
      3. Other family members
2. **Events that led to death of the child**

(Please document as the event were told by the respondent)

- 1. Could you tell me about the illness/events that led to her/his death?

**Hospitalisation period**

1. Now we would request you to tell us something about the period of hospitalisation for your child?

(Probe: hospital, ward, duration of stay, course of illness)

1. Could you please describe you overall experience about the hospital care?

(Probe: investigations, treatment given, involvement/ participation in decision making, satisfaction level, quality of care, cost of care)

1. Could you please describe your experience about communication by the various hospital staffs during hospital stay?

(Probe: who all communicated, who was the primary communicator, nurses, doctors, other staffs, frequency of communication, mode of communication, details, completeness of information, skill of communicator, consistency in communication)

**Death and post death period**

*We would like to know about the events around death and after death of your child.*

1. I your view what caused death of your child?

(Probe: who declared death, cause of death explained, preceding events before death, meeting with any senior doctor/member, attitude/body language/expression/skill of the communicator, expression of empathy/sympathy, language and simplicity, perceived completeness of information)

1. What was your and your family member’s reaction to the death of your child?

(Probe: what was the reaction of mother, father, other family members who were present, did someone try to console you or your family member)

1. How was your experience about the procedure after death till handover of the body to you?

(Probe: processes asked to follow, documentation and papers given, interaction and behaviour of the staffs, respect for religious norms, time taken, cost/payment, assistance from the staffs)

1. Who all from your family and community/neighbourhood were present at the hospital and how they supported you during this period?

(Probe: who informed them, who all came, what type support given)

**Knowing cause of death in detail – autopsy and MITS**

1. What was the cause of death of your child and what is your perception about the value of knowing the cause?

(Probe: knowledge about the cause of death, willingness to know the detailed cause, potential factors that led to the illness/death, potential impact on the other family members or next pregnancy)

*Many children die in our community due to various causes and in several instances the detail cause of death is not identified. We need to know the causes of death in detail. We want your views on the options of knowing the causes of death in detail.*

1. In your view, what more could have been done to know the exact cause of death and the reasons causing the disease?

(Probe: investigation, autopsy, any other)

1. Are you aware of any method for identifying the exact cause of death?

(Probe: autopsy, investigations, etc.)

*In several instances, doctors advise for autopsy/ post-mortem to find out the causes of death in children so that improvement in treatment and something can be done to prevent the deaths of children.*

1. What are your views about the autopsy/ post-mortem for identifying the cause of death?

(Probe: necessity, religious aspect, time needed, disfigurement, etc.)

*In some instances, autopsy/ post-mortem is not possible due to various reasons. To find out the exact cause of death and underlying disease, some tissue biopsy (collection of very small sample using needles, known as biopsy) and fluids (blood, urine, CSF, etc.) from the body of the child after they die, as done for investigation to identify and treat the diseases. But these samples are to be collected as soon as possible after death in the hospital.*

1. What are your views about collection of the tissue (like done for biopsy to diagnose diseases) and body fluid samples for identifying the cause of death?

(Probe: necessity, religious aspect, time needed, disfigurement, etc.)

1. How parents and family members can be approached for the tissue and fluid sampling?

(Probe: who should approach, whom, when and how should approach; what should be told and in what detail; should some picture of the method be used to explain)

1. In your view, what factors are important for acceptance of the tissue sampling by the parents and family members?

(Probe: desire to know the cause, trust, confidence on the health care provider)

**Decision making dynamics**

1. In routine day to day operation who takes decision about the major activities (purchases, treatment, spending money) about the family?

(Probe: involvement of the mother/women in the family, any other family member, consultation with any other member)

1. During the hospital stay who discussed with the doctors/ nurses to make the decision(s) about treatment and other procedures.

(Probe: father, mother, any other family member, did you/your spouse talk to someone in your family like elders or relatives to take critical decisions)

1. After death of your child, who decided about the cremation and other post-death activities and rituals?

(Probe: father, mother, any other family member; did you/your spouse talk to someone in your family like elders or relatives to take critical decisions)

**Summarisation**

1. Now, we are completing the interaction/ interview. Would you like to add anything?

Thank the respondent(s) for their critical contribution.

**Key Informant In-depth interview guide**

**Parent(s) of stillbirth**

1. **Basic demography**
   1. Demographic information
      1. Age
      2. Education
      3. Type of residence
      4. Religion
      5. Origin residence
   2. Family structure
      1. Composition- members
      2. Number of children
   3. Occupation
      1. Mother
      2. Father
      3. Other family members
2. **Events that led to the stillbirth**

Please let us know the course of pregnancy and events around the delivery/ termination.

(Please document as the event were told by the respondent)

- 1. Can you please let us know about the problems, illness and events immediately before the delivery?
  2. Where did you go or whom did you consult for any problem during the last pregnancy?

**Hospitalisation period**

1. Now we would request you to tell us something about the period of hospitalisation for the pregnancy, delivery or termination?

(Probe: hospital, ward, duration of stay, course of illness)

1. Could you please describe your overall experience about the hospital care?

(Probe: investigations, treatment given, involvement/ participation in decision making, satisfaction level, quality of care, cost of care)

1. Could you please describe your experience about communication by the various hospital staffs during hospital stay?

(Probe: who all communicated, who was the primary communicator, nurses, doctors, other staffs, frequency of communication, mode of communication, details, completeness of information, skill of communicator, consistency in communication)

**Stillbirth**

*We would like to know about the events around delivery and after that in the hospital.*

1. Could you please tell us about the declaration of the event/outcome (stillbirth)?

(Probe: who declared the outcome, cause explained, the body language and expression, preceding events before delivery, meeting with any senior doctor/member, attitude/body language/expression/skill of the communicator, expression of empathy/sympathy, language and simplicity, perceived completeness of information)

1. What was your and your family member’s reaction when you were told about the stillbirth?

(Probe: what was the reaction of mother, father, other family members who were present, did someone try to console you or your family member)

1. How was your experience about the procedure after death till handover of the body to you?

(Probe: processes asked to follow, documentation and papers given, interaction and behaviour of the staffs, respect for religious norms, time taken, cost/payment, assistance from the staffs)

1. Who all from your family and community/neighbourhood were present at the hospital and how they supported you during this period?

(Probe: who informed them, who all came, what type support given)

**Knowing cause of stillbirth in detail – autopsy and MITS**

*Many pregnancies in our community end with stillbirth due to various causes and in several instances the exact cause is not identified. We need to know the causes of stillbirth in detail. We want your views on the options of knowing the causes of stillbirth in detail.*

1. What was the cause of stillbirth mentioned to you and what is your perception about the value of knowing the cause?

(Probe: knowledge about the cause of stillbirth, willingness to know the detailed cause, potential factors that led to the illness/death, potential impact on the other family members or next pregnancy)

1. In your view, what more could have been done to know the exact cause of stillbirth and the reasons causing it?

(Probe: investigation, autopsy, any other)

1. Are you aware of any method for identifying the exact cause stillbirth?

(Probe: autopsy, investigations, etc.)

*In several instances, doctors advise for autopsy/ post-mortem to find out the causes of stillbirth so that appropriate action can be taken to prevent recurrence of such event.*

1. What are your views about the autopsy/ post-mortem for identifying the cause of stillbirth?

(Probe: necessity, religious aspect, time needed, disfigurement, etc.)

*In some instances, autopsy/ post-mortem is not possible due to various reasons. To find out the exact cause of stillbirth and underlying disease, some tissue biopsy (collection of very small sample using needles, known as biopsy) and fluids (blood, urine, CSF, etc.) from the body, as done for investigation to identify and treat the diseases. But these samples are to be collected as soon as possible.*

1. What are your views about collection of the tissue (like done for biopsy to diagnose diseases) and body fluid samples for identifying the cause of stillbirth?

(Probe: necessity, religious aspect, time needed, disfigurement, etc.)

1. How parents and family members can be approached for the tissue and fluid sampling?

(Probe: who should approach, whom, when and how should approach; what should be told and in what detail; should some picture of the method be used to explain)

1. In your view, what factors are important for acceptance of the tissue sampling by the parents and family members?

(Probe: desire to know the cause, trust, confidence on the health care provider)

**Decision making dynamics**

1. In routine day to day operation who takes decision about the major activities (purchases, treatment, spending money) about the family?

(Probe: involvement of the mother/women in the family, any other family member, consultation with any other member)

1. During the hospital stay who discussed with the doctors/ nurses to make the decision(s) about treatment and other procedures.

(Probe: father, mother, any other family member, did you/your spouse talk to someone in your family like elders or relatives to take critical decisions)

1. After the delivery, who decided about the burial and other post-death activities and rituals?

(Probe: father, mother, any other family member; did you/your spouse talk to someone in your family like elders or relatives to take critical decisions)

**Summarisation**

1. Now, we are completing the interaction/ interview. Would you like to add anything?

Thank the respondent(s) for their critical contribution.

**Key Informant In-depth interview guide**

**Community Informants**

1. **Basic demography**
   1. Designation/Role
   2. Occupation
   3. Period of stay at this place
2. **Care seeking practices**
   1. Where do people from your community/ locality usually go or whom do you consult for healthcare (all types of patients/illnesses)?
   2. Which all health facilities are usually accessed? Which ones are mostly accessed?
   3. Which health providers are usually accessed? Which ones are mostly trusted?
3. Where do people from your community/ locality usually go or whom do you consult during illness of children and newborn?
   1. Which ones are usually accessed for non-critical illnesses and why?
   2. Which ones are usually accessed for critical or severe illnesses and why?
4. Where do people from your community/ locality usually go or whom do you consult during pregnancy and delivery?
   1. Which ones are usually accessed for routine pregnancy check-ups?
   2. Which ones are usually accessed for delivery or pregnancy with complications?

**Illnesses and causes of death**

1. In your view, what are the common illnesses and causes of child death in your locality?
2. *We are trying to identify the causes of death in children, so that something can be done to prevent and reduce the number of deaths in children.*

In your view, would this information be valuable?

(If valuable/ useful, why? If not, why not?)

**Knowing cause death and implications of procedures**

*In some cases the children die before even the exact cause of illness and death is identified/ investigated. In such cases, doctors suggest autopsy/ post-mortem to identify the cause(s) of death.*

1. How autopsy/post-mortem is viewed by you and community members for knowing the cause of death?

(Probe: explore the views, concerns and believes in the community regarding autopsy)

1. *In some instances, autopsy/ post-mortem is not possible due to various reasons. To find out the exact cause of death and underlying disease, some tissue biopsy (collection of very small sample using needles) and fluids (blood, urine, etc.) from the body of the child after they die, as done for investigation. But these samples are to be collected as soon as possible after death.*

How do you view minimal tissue sampling procedure as a method for knowing the cause of death?

(Probe: explore the views, concerns and believes, religious aspects, comparison with autopsy)

1. How the parents/ families are likely to view the minimal tissue sampling procedure as a method for knowing the cause of death?

(Probe: anticipated views, concerns and believes, religious aspects, rituals, comparison with autopsy)

1. How the parents/families of a child/newborn who has died, should be approached by the hospital doctors/staffs for the minimal tissue sampling procedure?

(Probe: who, when and how and whom should approach, who can be key influencer)

1. In your view, what factors are important for acceptance of the minimal tissue sampling by the parents and family members?

(Probe: desire to know the cause, trust, confidence on the health care provider, who can be key influencer)

1. In your view, what would be the most suitable method(s) of educating the community about the minimal tissue sampling?

(Probe: medium, methods, place and target audience)

1. In your view, what are the challenges for conducting the minimal tissue sampling at the hospitals?

(Probe: acceptance by community and families, religious aspects, burial/cremation related)

1. In your view, how the community leaders/influencers like you can contribute/ participate in such activity?

(Probe: who or what is it, what did you do in this context)

1. Any other specific comments/ observations.

Thank the respondent(s) for their critical contribution.

**Key Informant In-depth interview guide**

**Religious Leader/ Burial Site representative**

1. **Basic demography**
   1. Religion
   2. Designation
   3. Total years of service
   4. Total years in current position

**Death and related practices**

1. Please describe the rituals and practices followed around and after death (Please describe these day-wise and duration).

(Probe: for adults, children, neonates aged < 1 month, still birth and abortions)

1. Please describe the differences in rituals and practices according to the place of death and natural versus premature death like a child and at the time of delivery/birth.

(Probe: death at home versus death at hospital)

1. In your view, what happens to the spirit, when a child/newborn dies? What is usually done by the family and/or community to help this happen?

(Probe: what happens, what things are done, what are not done, what happens if they are not done)

1. Is there any social stigma or taboo related to family or woman in relation to her child’s death or stillbirth?

(Probe: blame, spiritual factors, effect on family or children or subsequent pregnancy)

**Knowing cause death and implications of procedures**

1. In your view, how important is to know the cause of death?

(Probe: How this can help the parents/family and others? How is viewed in the community?)

1. What are the beliefs about post mortem (or autopsy) in your religion?

(Probe: acceptance, religious and spiritual concerns)

1. After a post-mortem is done, are there any modifications in the rituals done in the funeral process?

(Probe: body preparation, mode of burial/cremation, post burial/cremation practices, related cost implications)

*In some instances, autopsy is not possible due to various reasons. To find out the exact cause of death and underlying disease, some tissue biopsy (collection of very small sample using needles) and fluids (blood, urine, etc.) from the body of the child after they die, as done for investigation. But these samples are to be collected as soon as possible after death. This does not cause any disfigurement of the body.*

1. What are your views about collection of the tissue and body fluid samples for identifying the cause of death?

(Probe: necessity, religious aspect, time needed, etc.)

1. How parents and family members can be approached for the tissue sampling?

(Probe: who, when and how should approach and whom)

1. In your view, what factors are important for acceptance of the tissue sampling by the parents and family members?

(Probe: desire to know the cause, trust, confidence on the health care provider)

1. In your view, is there any modifications in the rituals done in the funeral process needed with the minimal tissue sampling procedure?

(Probe: body preparation, mode of burial/cremation, post burial/cremation practices, related cost implications)

1. Any other opinion/ suggestions.

Thank the respondent for his critical contribution.

**Focus Group Discussion guide**

Category of stakeholders: (Husbands/Fathers; Women with children <5 years age; Father-in-laws and Mother-in-laws)

Number of participants:

Age of the participants:

Occupation of the participants:

**Illnesses, death and care seeking practices**

*You are aware that several of the children in the area/ community fall ill/become sick and some of them die.*

1. In your opinion, how and why the children fall ill/ become sick? Why some of them die?

(Probe: the microbial causes, rituals, religious aspects, spiritual and other issues)

1. Where do people from your community/ locality usually go or whom do you consult for healthcare (all types of patients/illnesses)?
   1. Which all health facilities are usually accessed? Which ones are mostly accessed?
   2. Which health providers are usually accessed? Which ones are mostly trusted?
2. Where do people from your community/ locality usually go or whom do you consult during illness of children and newborn?
   1. Which ones are usually accessed for non-critical illnesses and why?
   2. Which ones are usually accessed for critical or severe illnesses and why?

**Pregnancy, outcome and care seeking practices**

*You are aware that several of the pregnancies in the area/community terminate with stillbirth (death during delivery) or intrauterine death (death inside womb).*

1. In your opinion, how and why some of the pregnancies terminate with stillbirth or abortion?

(Probe: care, diseases, rituals, religious aspects, spiritual and other issues)

1. Where do people from your community/ locality usually go or whom do you consult during pregnancy and delivery?
   1. Which ones are usually accessed for routine pregnancy check-ups?
   2. Which ones are usually accessed for delivery or pregnancy with complications?
2. What are the rituals and practices observed by the family and community when a child dies?
   1. What practices are followed when a child dies before completion of first month (or 21 days)?
   2. What practices are followed when a child dies before fifth birth day?
   3. What practices are followed when a child dies during delivery and declared born dead?
3. How does the parents and family members cope with the death of their child?

(Probe: coping by mother/pregnant woman, father/ husband, family members, support from relatives and community)

**Causes of death and knowing the cause**

1. In your view, what are the common illnesses and causes of child death in your locality?
2. In your view, what are the common causes of stillbirth and intrauterine death in your locality?

*We are trying to identify the causes of death in children and stillbirths, so that something can be done to prevent and reduce the number of deaths in children and stillbirths.*

1. In your view, would this information be valuable?

(Probe: If valuable/ useful, why? If not, why not?)

*In some cases the children die before even the exact cause of illness and death is identified/ investigated. In such cases, doctors suggest autopsy/ post-mortem to identify the cause(s) of death.* Similarly for the stillbirths and intrauterine deaths*, doctors suggest autopsy/post-mortem of the baby to identify the cause(s) of death.*

1. How autopsy/post-mortem is viewed by you and community members for knowing the cause of death?

(Probe: views, concerns and believes in the community regarding autopsy)

*In some instances, autopsy/ post-mortem is not possible due to various reasons. To find out the exact cause of death and underlying disease, some tissue biopsy (collection of very small sample using needles) and fluids (blood, urine, etc.) from the body of the child after they die, as done for investigation. But these samples are to be collected as soon as possible after death.*

1. How do you view minimal tissue sampling procedure as a method for knowing the cause of death?

(Probe: views, concerns and believes, religious aspects, comparison with autopsy)

1. How the parents/ families are likely to view the minimal tissue sampling procedure as a method for knowing the cause of death?

(Probe: anticipated views, concerns and believes, religious aspects, rituals, comparison with autopsy)

1. How the parents/families of a child/newborn who has died, should be approached by the hospital doctors/staffs for the minimal tissue sampling procedure?

(Probe: who, when and how and whom should approach, who can be key influencer)

1. In your view, what factors are important for acceptance of the minimal tissue sampling by the parents and family members?

(Probe: desire to know the cause, trust, confidence on the health care provider, who can be key influencer)

1. In your view, what would be the most suitable method(s) of educating the community about the minimal tissue sampling?

(Probe: medium, place and target audience)

1. In your view, how the community leaders/influencers like you can contribute/ participate in such activity?

(Probe: who or what is it, what did you do in this context)

1. Any other specific comments/ observations.
